# Artificial intelligence-based photographic detection of pink esthetic score attributes using a hybrid deep learning segmentation pipeline: a method development study

**DOI:** 10.1038/s41598-026-62229-4

**Published:** 2026-08-01

**Authors:** Mihad Ibrahim, Ghada Gamal Adayil, Nouran Hany, Nourhan Emad, Manal Mohamed Hosny

**Affiliations:** 1 Department of Periodontology, Faculty of Dentistry, Cairo University, 11 Saray Street, Almanial, Cairo, Egypt; 2 Faculty of Dentistry, Cairo University, Cairo, Egypt; 3 Computer Vision Engineer at Cyshield Egypt, Cairo, Egypt; 4 Computer Vision Specialist Engineer and Team Lead at Cyshield Egypt, Cairo, Egypt

**Keywords:** Artificial intelligence, Deep learning, Dental implants, Esthetics, Computer-aided diagnosis, Computational biology and bioinformatics, Health care, Mathematics and computing, Medical research

## Abstract

This study aimed to develop and internally validate a novel anatomy-driven artificial intelligence (AI) system for automated postoperative Pink Esthetic Score (PES) evaluation from intraoral photographs. Unlike most existing AI approaches, which rely on end-to-end prediction, the proposed pipeline derives PES attributes from anatomically grounded measurements. A hybrid analytical pipeline integrating instance segmentation and rule-based measurement was developed. Tooth crown segmentation was performed using Mask R-CNN, while gingival segmentation was performed using YOLOv11. Anatomical features were extracted from the segmented structures and converted into ordinal PES attributes through data-driven threshold calibration derived from the training dataset. The reference standard consisted of independent expert scoring of 82 postoperative intraoral photographs. Diagnostic accuracy, confusion matrix analyses, and regression metrics were calculated to evaluate agreement between automated and expert assessments. The hybrid segmentation-based system achieved accuracies of 91.5% for the mesial papilla, 85.4% for the distal papilla, 86.6% for the gingival margin, and 82.9% for gingival color assessment. Exact agreement between automated and expert total PES scores was observed in 57.3% of cases, increasing to 79.3% when a clinically relevant tolerance of ± 1 PES point was applied. Regression analysis demonstrated a mean absolute error of 0.76, a root mean squared error of 1.35, and an R² value of 0.49. An anatomy-driven segmentation framework may provide a transparent and reproducible approach for automated photographic PES attribute assessment in research settings.

**Supplementary Information:**

The online version contains supplementary material available at 10.1038/s41598-026-62229-4.

## Introduction

Esthetic outcomes have become a central determinant of success in anterior implant therapy, where peri-implant soft-tissue appearance is closely tied to patient satisfaction and is a frequent indication for soft-tissue augmentation^1^. Reflecting this, recent consensus work in periodontology and implant dentistry has emphasised standardised, objective assessment of esthetic and patient-reported outcomes^2^.

Numerous esthetic indices have been proposed over the past two to three decades to objectively evaluate peri-implant soft-tissue and crown esthetics. The Pink Esthetic Score (PES)^3^ focuses on peri-implant soft tissue; the Implant Crown Aesthetic Index (ICAI)^4^ additionally scores the restoration; and the combined Pink and White Esthetic Score (PES/WES)^5^ integrates soft-tissue and crown criteria. Further indices including the Complex Esthetic Index, the Implant Aesthetic Score, the Subjective Esthetic Score, and the Rompen Index differ in the parameters scored, their weighting of hard- and soft-tissue components, and their reproducibility^6,7^. Among these, the PES, alone or combined with the WES, has become the most widely adopted index in clinical research^8,9^, owing to its focus on clinically meaningful peri-implant soft-tissue parameters and its straightforward ordinal scoring, which facilitates cross-study comparison. Despite demonstrating acceptable reliability in clinical studies, variability in scoring has been reported. It may be influenced by examiner experience and subspecialty background^10,11^. Accordingly, strategies aiming to improve scoring consistency and reproducibility may enhance the reliability of peri-implant esthetic assessment in research settings.

Artificial intelligence (AI), particularly through advances in deep learning–based image analysis, is increasingly applied across fields of dentistry to improve diagnostic accuracy, efficiency, and consistency in periodontal and implant-related applications^12–14^. A previous deep learning model approached PES assessment through direct prediction of the overall composite score^15^. Yet, the PES represents a composite index derived from the evaluation of distinct anatomical features; hence, an accurate assessment may necessitate attribute-level, feature-driven analysis rather than holistic score prediction.

The current study aims to develop and internally validate a novel deep learning model for automated assessment of peri-implant esthetic attributes derived from the PES framework, in order to improve standardization and reproducibility in research settings.

## Materials and methods

### Study design and ethical approval

This study was designed as a cross-sectional diagnostic accuracy validation study and was conducted in accordance with the Standards for Reporting Diagnostic Accuracy Studies guidelines for Artificial Intelligence (STARD-AI)^16^. Ethical approval was obtained from the Research Ethics Committee of the Faculty of Dentistry, Cairo University (approval number 06–25). The research was conducted in compliance with the Declaration of Helsinki. Informed consent for the use of clinical photographs for research purposes was obtained from all participants. The AI-based photographic PES assessment system constituted the index test, and expert PES scoring served as the reference standard (Sect. Reference standard).

### Study population and dataset curation

Postoperative records from the Department of Periodontology, Cairo University, were retrospectively reviewed for cases involving single implant-supported restorations placed between January 2020 and June 2025. Inclusion criteria were: the presence of a single implant-supported crown in the maxillary esthetic region, availability of two natural adjacent teeth to enable papilla assessment, and high-quality intraoral photographs with unobstructed visualization of peri-implant soft tissues.

**Fig. 1:**
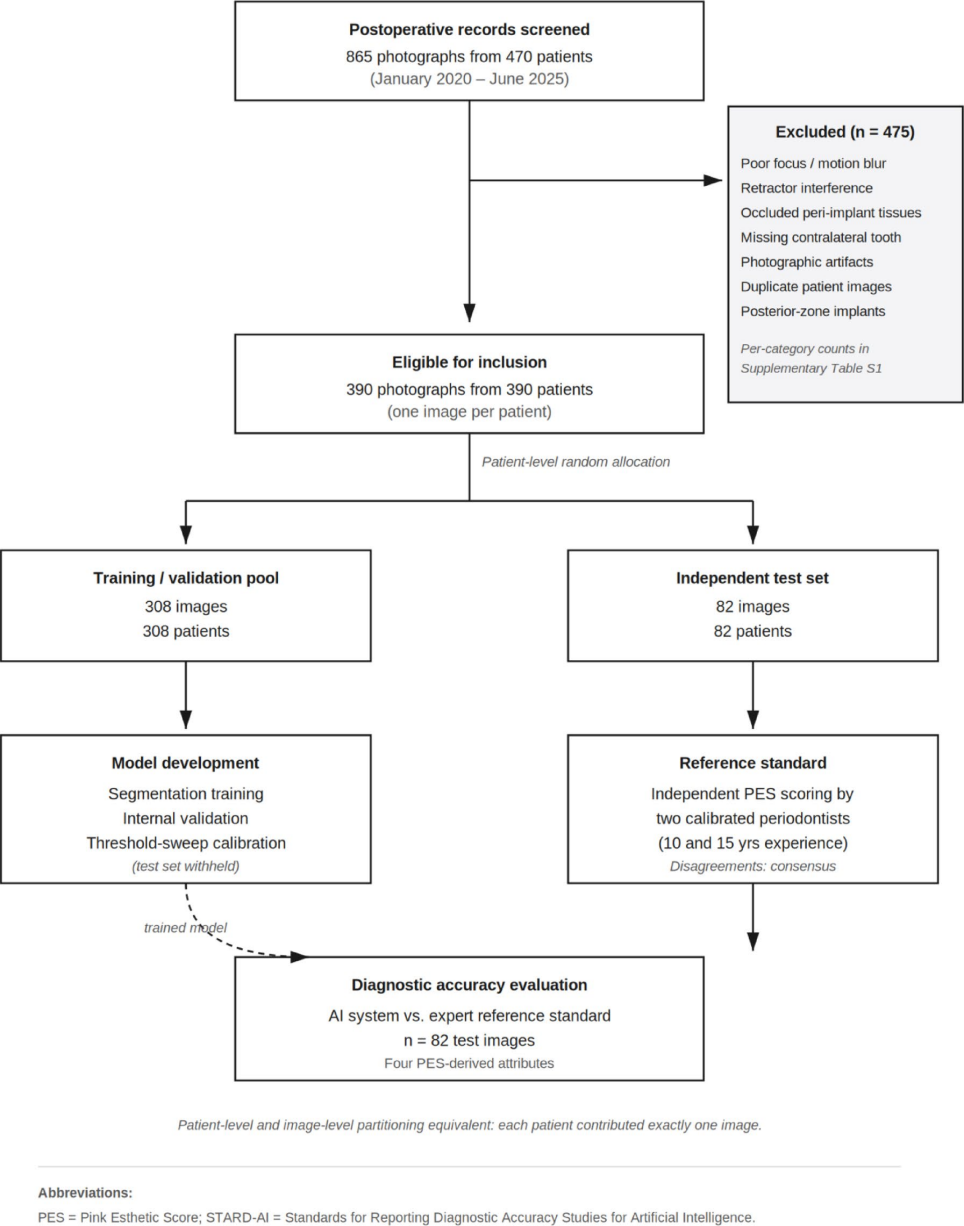
STARD-AI flow of photograph selection, patient-level allocation, and diagnostic-accuracy evaluation.

A total of 865 photographs from 470 patients were screened; 475 photographs were excluded, with category-wise counts reported in Table 2. The remaining 390 photographs originated from 390 unique patients (one image per patient), eliminating within-patient clustering by construction. Photographs displaying mild acquisition imperfections or clinically observable peri-implant inflammation were retained when peri-implant anatomy remained clearly visible, in order to reflect realistic intraoral acquisition conditions. The data curation workflow is summarized in Fig. 1; Table 1.

**Table 1:** Cohort characteristics of the included patients (*n* = 390).

Variable	Definition	Value
Number of patients	Unique patients included after curation	390
Age	Mean ± SD (range)	44.3 ± 7.5 years (range not provided)
Sex distribution	Male n (%)/Female n (%)	120 (30.8%) male/270 (69.2%) female
Implant site	FDI tooth number distribution	11 (*n* = 77), 21 (*n* = 73), 12 (*n* = 38), 22 (*n* = 30), 13 (*n* = 45), 23 (*n* = 21), 14 (*n* = 32), 24 (*n* = 35), 15 (*n* = 24), 25 (*n* = 15)
Time since prosthetic delivery	Months post-restoration (median, IQR)	21 months (13.5–28.5)
Soft-tissue status	Healthy/mild/moderate/severe inflammation n (%)	40 (10.3%) healthy, 179 (45.9%) mild, 146 (37.4%) moderate, 25 (6.4%) severe
Peri-implant condition	Healthy/mucositis/peri-implantitis n (%)	82 (21.0%) healthy, 163 (41.7%) mucositis, 145 (37.1%) peri-implantitis
Image acquisition quality	Good/acceptable/mild imperfections n (%)	36 (9.3%) good, 163 (41.7%) acceptable, 191 (48.9%) mild imperfections
Challenging features	Saliva, shadows, highlights, blur, etc.	191 photos with challenging features; 88 with multiple artifacts

Values are n (%) unless otherwise stated; implant sites are given as FDI tooth numbers. Soft-tissue status, peri-implant condition, and image acquisition quality were categorised during image curation.

**Table 2:** Reasons for exclusion of intraoral photographs from the Cairo University gingival dataset (475 of 865 screened).

Exclusion category	*n* excluded
Poor focus or motion blur	60
Retractor interference obscuring peri-implant tissues	10
Occluded peri-implant soft tissues (lip, tongue, saliva pooling)	45
Missing or non-visualized contralateral reference tooth	50
Other photographic artifacts (specular blow-out, severe color cast)	15
Duplicate images from the same patient (one representative image retained)	95
Implants outside the maxillary esthetic zone (posterior sites)	200
Total excluded	**475**

Categories are non-overlapping; each excluded photograph was assigned to a single primary reason. The 390 included photographs originated from 390 unique patients, with one image per patient.

Dataset partitioning was performed strictly at the patient level by simple random sampling. The 390 patient-image pairs were allocated to a training/validation pool (*n* = 308) and an independent test set (*n* = 82); no patient appears in more than one subset. Because each patient contributed exactly one image, patient-level and image-level partitioning were equivalent. All images were anonymized before analysis.

The tooth crown segmentation model used a separate, externally sourced training dataset: the Roboflow “Teether” dataset (*n* = 1,966 intraoral photographs), acquired across heterogeneous sources, camera systems, and patient populations. This dataset shares no images or patient identifiers with the Cairo University dataset or the 82-image independent test set.

### Reference standard

The reference standard for esthetic evaluation was based on peri-implant esthetic attributes derived from the modified five-point PES framework proposed by Belser et al.

Four of the five PES attributes were assessed: mesial papilla, distal papilla, level of the soft-tissue margin, and soft-tissue color. The contour attribute was not included due to the limitations of standardized two-dimensional photographic records in reliably capturing three-dimensional soft tissue convexity. Each attribute was scored on a three-point scale (0 = poor, 1 = moderate, 2 = ideal), yielding a maximum total score of 8.

Reference standard scoring was performed by the first author (M.I.) and a senior periodontist co-author (G.A), applying the Belser et al. (2009) criteria. Each test image was scored independently by both scorers in a structured Microsoft Excel spreadsheet, blinded to AI-generated outputs, to patient identifiers (only sequential image codes were shown), and to time since treatment. AI predictions were generated only after reference standard scoring was complete. Discrepant cases were resolved through consensus discussion with reference to the Belser criteria.

### Annotation

#### In-house gingival dataset

Gingival segmentation training masks for the in-house Cairo University dataset (*n* = 308 training/validation images) were generated by the first author (M.I.) as the single annotator, using manual polygon annotation in the cloud-hosted CVAT platform (https://app.cvat.ai), accessed during the study period. Four polygon classes were annotated on the full image: marginal gingiva, mesial papilla, distal papilla, and alveolar mucosa. Polygons were exported in COCO instance-segmentation format. Written annotation guidelines were developed prior to annotation by consensus between the annotator, the senior periodontology supervisors, and the AI development team; the guidelines were applied during a 20-image calibration step (using images not subsequently included in either the training/validation pool or the independent test set) and throughout dataset annotation.

#### External tooth-crown dataset

Tooth crown segmentation training masks (*n* = 1,966) from the Roboflow “Teether” dataset were generated by the dataset’s original contributors using polygon annotation in COCO instance-segmentation format per Roboflow Universe conventions. The annotation protocol for this external dataset is governed by its original publishers; no modification or re-annotation was performed for the present study.

### Segmentation models

#### Preliminary classification experiments

As an initial strategy, direct image classification models were developed using MobileNetV2 and ResNet-50 architectures with transfer learning to predict total PES-derived scores from whole intraoral photographs. The limited predictive performance achieved by these models prompted the development of a segmentation-based, anatomy-driven pipeline for attribute-level analysis.

#### Backbone selection experiment

To assess how the choice of segmentation architecture influences automated PES scoring, different backbone models were tested within two instance segmentation frameworks: Mask R-CNN (Detectron2) and YOLOv11 (Ultralytics). Four pipeline configurations were constructed by combining these backbones for tooth crown and gingival segmentation tasks. For each configuration, identical downstream anatomical processing and rule-based scoring procedures were applied, including central tooth identification, contralateral pairing, papilla triangle construction, gingival margin height extraction, and PES-derived attribute computation. All pipelines were evaluated using the independent 82-image test dataset under a predefined evaluation protocol.

#### Final architectures

**Fig. 2:**
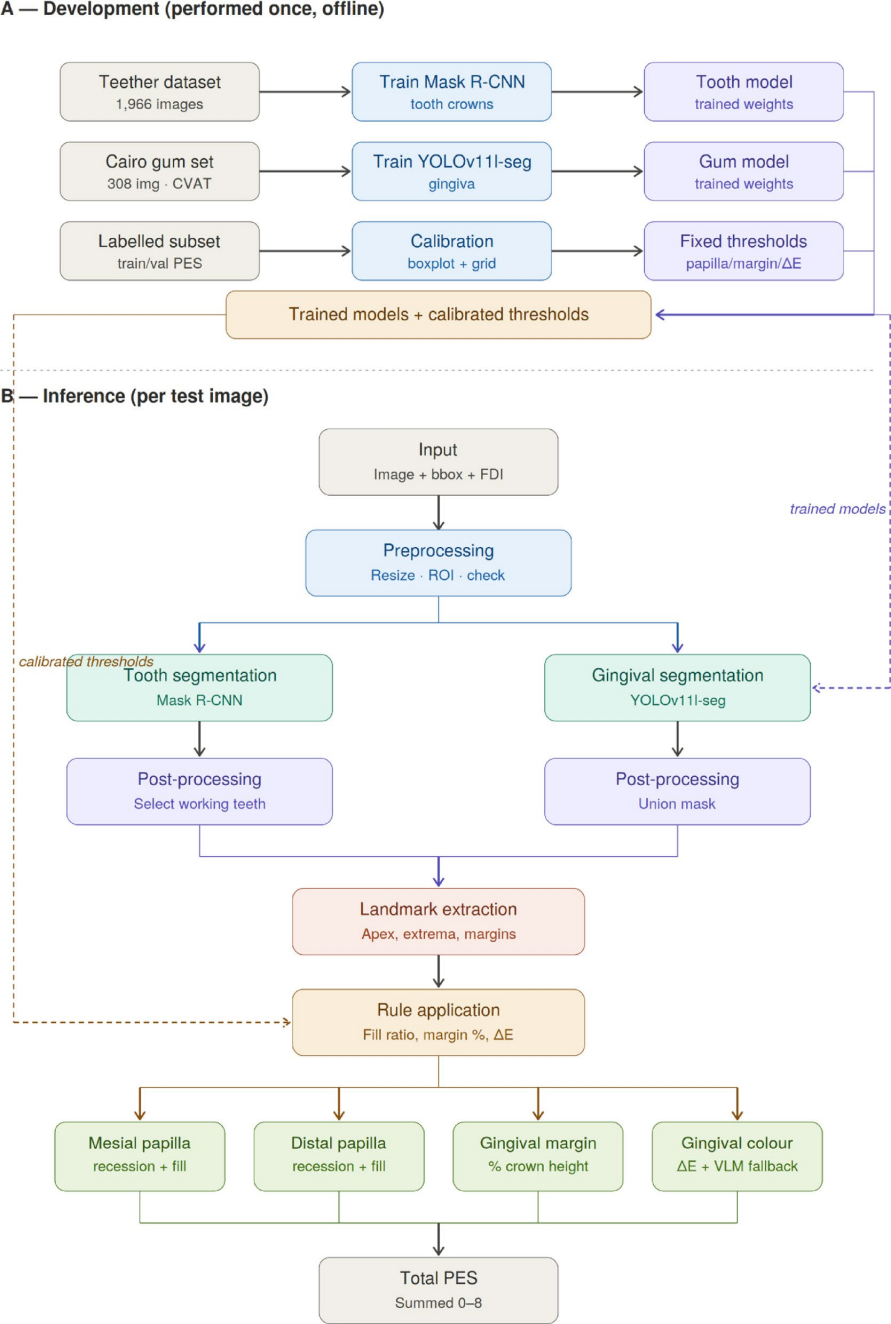
Overview of the anatomy-driven PES pipeline. (**A**) Development (performed once, offline): the tooth-crown segmentation model (Mask R-CNN) was trained on the external Teether dataset (*n* = 1,966 images); the gingival segmentation model (YOLO11l-seg) was trained on 308 Cairo University photographs annotated in CVAT; and scoring thresholds were fixed on a labelled train/validation subset using box-plot midpoints followed by grid search. (**B**) Inference (per test image): each image, with its bounding box and FDI label, is preprocessed (resize, ROI selection, quality check) and passed in parallel to tooth and gingival segmentation. After post-processing (working-tooth selection and gingival mask union), anatomical landmarks (papilla apex, gingival extrema, margin position) are extracted, and rule-based logic derives the four PES attributes — mesial papilla, distal papilla, gingival margin, and gingival color — which sum to the total PES (0–8).

**Fig. 3:**
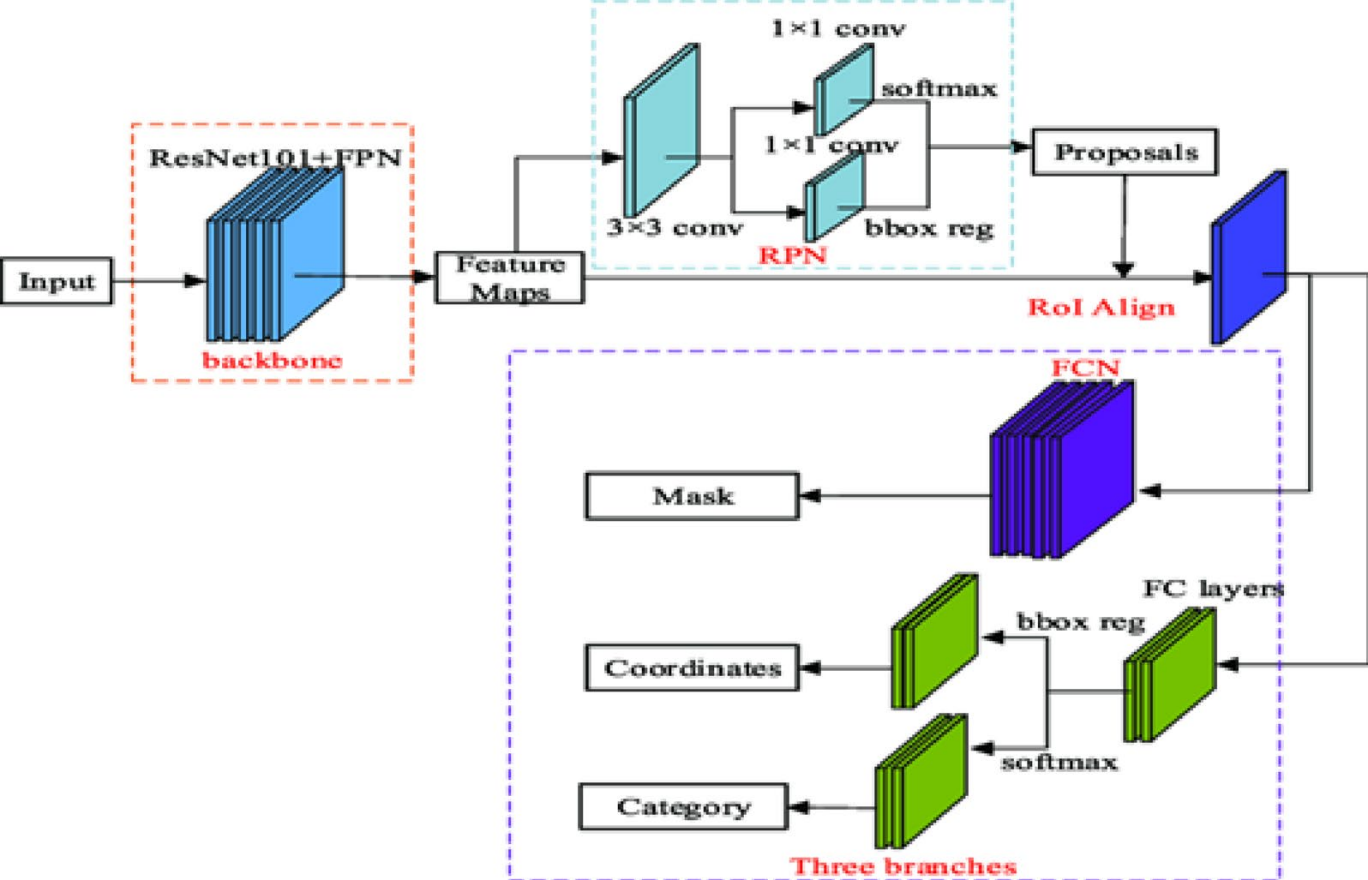
Mask R-CNN architecture for tooth-crown segmentation. The model comprises a ResNet-50–FPN backbone for feature extraction, a region proposal network (RPN) generating candidate regions, RoI Align for spatial feature pooling, and three parallel output branches: a fully convolutional network (FCN) producing the segmentation mask, a bounding-box regression branch, and a classification branch. The model was initialised from COCO-pretrained weights.

**Fig. 4:**
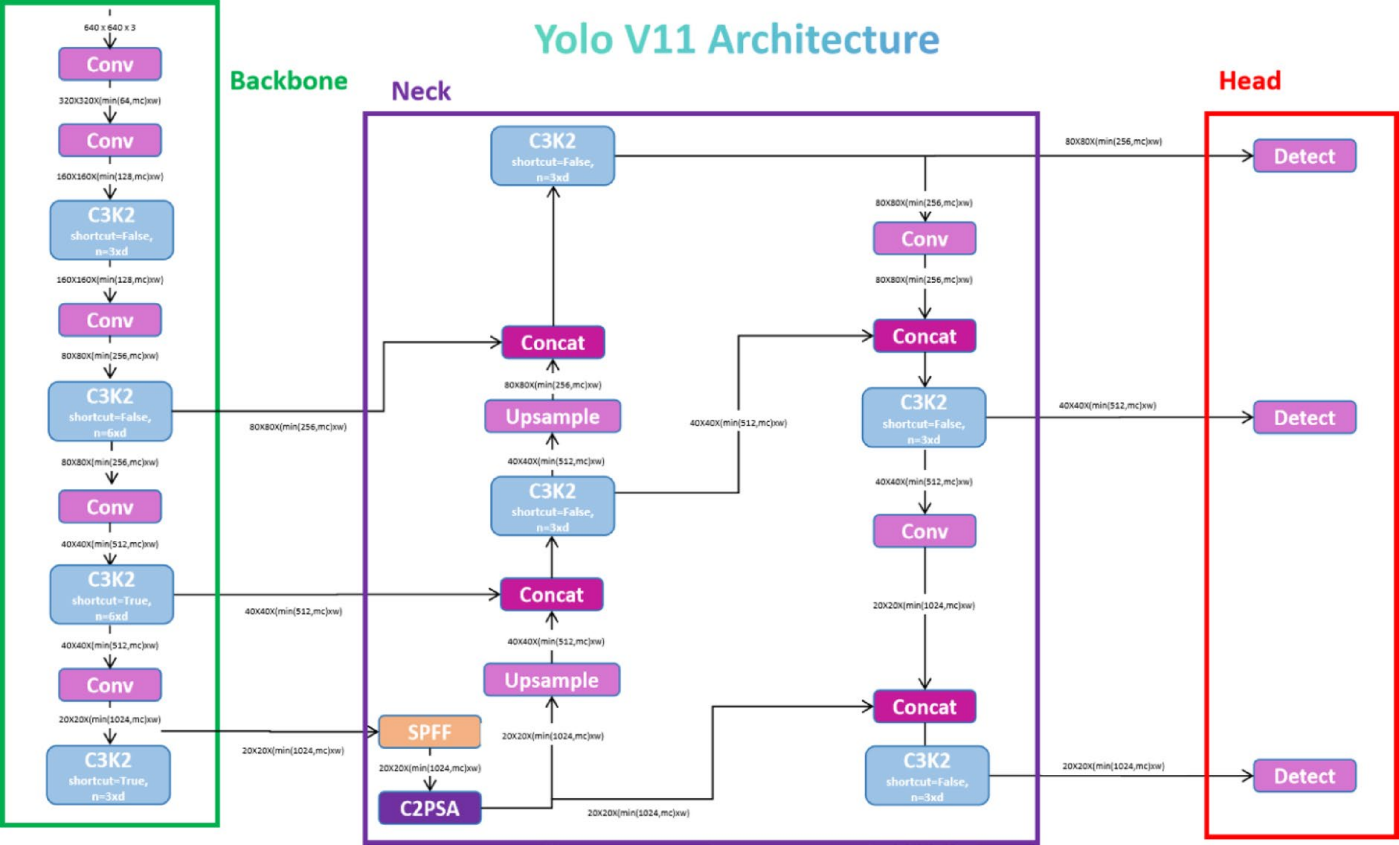
YOLO11l-seg architecture for gingival segmentation. The network is organised into backbone, neck, and head. Input photographs (640 × 640 × 3) pass through successive convolutional and C3K2 blocks, an SPPF module, and a C2PSA attention block; the neck performs multi-scale feature aggregation via upsampling and concatenation, and the multi-scale heads output the gingival segmentation at three spatial resolutions. The model was initialised from COCO-pretrained weights and fine-tuned end-to-end on the Cairo University gingival dataset.

Following the structured comparison, the final integrated system employed Mask R-CNN with a ResNet-50 Feature Pyramid Network (FPN) backbone, implemented via Detectron2, for tooth crown segmentation and YOLO11l-seg (Ultralytics, Large variant; approximately 27.6 million parameters) for gingival segmentation. This hybrid configuration was used for automated extraction of PES-derived attributes in the complete assessment framework. The integrated project pipeline is displayed in Fig. 2; the tooth crown segmentation pipeline in Fig. 3; and the gingival segmentation pipeline in Fig. 4.

### Training

#### Data augmentation

Data augmentation differed between the two segmentation models. For tooth crown segmentation (Mask R-CNN), training used Detectron2 framework defaults, including multi-scale resizing. For gingival segmentation (YOLO11l-seg), online data augmentation was enabled during training to improve robustness against intraoral photographic variability. Augmentation parameters included geometric translation of ± 10% of image size (translate = 0.1) and isotropic scaling of ± 50% (scale = 0.5). Shear and perspective transformations were disabled (shear = 0, perspective = 0). Horizontal flipping was applied with a probability of 50% (fliplr = 0.5); vertical flipping was disabled (flipud = 0.0). Color augmentation was performed in HSV space using hue jitter ± 1.5% (hsv_h = 0.015), saturation jitter ± 70% (hsv_s = 0.7), and brightness jitter ± 40% (hsv_v = 0.4). Mosaic augmentation was enabled (mosaic = 1.0) and automatically disabled during the final 10 epochs (close_mosaic = 10). MixUp and copy–paste augmentation were disabled (mixup = 0.0, copy_paste = 0.0). Random erasing (erasing = 0.4) was additionally applied. Rotation, shear, perspective transformation, and vertical flipping were deliberately disabled to preserve the vertically oriented anatomical relationships underlying PES computation. MixUp and copy–paste augmentation were excluded because they generate anatomically implausible tissue blending or artificial relocation of gingival structures without a realistic clinical analogue.

#### Training configuration

##### Tooth crown segmentation (Mask R-CNN, Detectron2)

The tooth-crown model was a Mask R-CNN with a ResNet-50 + Feature Pyramid Network backbone, implemented in Detectron2 (v0.6) and initialised from COCO-pretrained model-zoo weights. Under Detectron2’s default configuration, the backbone stem and first residual stage (res2) were frozen (FREEZE_AT = 2), and res3–res5, the FPN, the RPN, and the detection/segmentation heads were fine-tuned. Training used Detectron2’s default SGD optimiser and learning-rate schedule (momentum 0.9, weight decay 1 × 10⁻⁴, linear warm-up) with a batch size of 16, for 2,998 iterations (≈ 25.5 epochs over the 1,881-image training set). Default augmentation comprised multi-scale ResizeShortestEdge resizing (shorter edge sampled from {640, 672, 704, 736, 768, 800} px; maximum longer edge 1333 px; bilinear interpolation) and random horizontal flipping during training; at inference, a fixed shorter edge of 800 px (maximum 1333 px) was used. The Roboflow “Teether” dataset was partitioned into training (*n* = 1,881; 96%), validation (*n* = 49; 2%), and internal test (*n* = 36; 2%) subsets.

##### Gingival segmentation (YOLO11l-seg, Ultralytics 8.3.65)

The gingival model was YOLO11l-seg, initialised from COCO-pretrained weights (yolo11l-seg.pt); all layers, backbone, neck (PANet), and segmentation head, were fine-tuned end-to-end, with no layers frozen. Training ran for up to 100 epochs with early stopping (patience = 10; model selection on the highest validation segmentation mAP), which guarded against memorisation and unnecessary optimisation after convergence, using a batch size of 16 and SGD with momentum 0.937. The initial learning rate was 0.01 (lr0 = 0.01) with a final learning-rate fraction of 0.01 (lrf = 0.01) and weight decay 5 × 10⁻⁴; a 3-epoch warm-up phase (warmup_momentum = 0.8, warm-up bias learning rate = 0.1) stabilised early training. Strong data augmentation served as the primary anti-overfitting mechanism, comprising geometric (translation, scaling), photometric (HSV jitter), spatial (horizontal flip), and structural (mosaic, random erasing) transforms together with a RandAugment policy, simulating camera, lighting, and framing variability to increase the effective dataset size. The Ultralytics framework letterboxed input photographs (640 × 480 after the standardised preprocessing chain) to a 640 × 640 tensor (bilinear interpolation; pad value 114). A fixed random seed (0) with PyTorch/cuDNN deterministic mode enabled ensured run-to-run reproducibility. The in-house Cairo University gingival dataset ;separate from the Teether dataset above was partitioned into training (*n* = 296; 96%), validation (*n* = 6; 2%), and internal test (*n* = 6; 2%) subsets.

#### Overfitting control and reproducibility

Across both models, generalisation was supported by the combination of COCO transfer learning, a dedicated validation set for unbiased monitoring, principled training-length selection (early stopping for the YOLO model; a fixed 2,998-iteration budget for the Mask R-CNN model), optimisation regularisation, and, for the gingival model, strong data augmentation; the held-out internal test sets were excluded from all training and validation. Analysis of the YOLO11l-seg training logs showed smooth, near-monotonic decreases in both training and validation loss with no divergence between the curves, consistent with well-generalised training and no evidence of overfitting.

##### Computational environment

Model training and inference were both performed on a workstation equipped with an Intel Core i9-14900 K CPU and an NVIDIA GeForce RTX 4090 GPU (24 GB VRAM), running CUDA 12.1. All experiments were implemented in Python (v3.11.9) using PyTorch (v2.5.1) as the base deep-learning framework. Inference-time geometric and color computations used OpenCV 4.10.0.84 for image preprocessing and contour extraction, scikit-image (v0.24.0), and scikit-learn (v1.6.1) for image operations and confusion-matrix computation. The Qwen 2.5 vision–language model used for ambiguous-color-case fallback was run under fixed inference parameters and required GPU hardware with ≥ 24 GB VRAM. Approximate training times were 20–30 min for the Yolo11-lSeg model and 1–3 h for Mask R-CNN.

#### Threshold calibration

To convert continuous anatomical measurements into ordinal PES categories 0–2, thresholds were determined through a two-stage procedure on a PES-labeled subset of the training/validation pool. In the first stage, initial threshold values were defined at the midpoints between adjacent class medians, identified by box-plot distribution analysis of measurement values grouped by expert-assigned PES scores. In the second stage, these initial values were refined by exhaustive grid search around the initialization, optimizing a combined objective of weighted F1 score (to handle class imbalance) and overall accuracy against the calibration-subset PES labels. Once selected, threshold values were fixed.

#### Test-set isolation

The 82-image independent test dataset was fed to the trained pipeline only at inference time for the single, pre-specified final evaluation. It was not used during model training, validation, hyperparameter tuning, or threshold calibration at any stage, ensuring no data leakage between calibration and evaluation.

### Inference pipeline

#### Image preprocessing and segmentation

Before inference and PES scoring, all images underwent standardized preprocessing: (1) loading in BGR color space; (2) resizing to 640 × 480 pixels using bilinear interpolation; (3) detection of the clinician-drawn green bounding rectangle via HSV thresholding (H ∈ [40, 85], S > 80, V > 80) for region-of-interest localization.No color normalization, per-channel intensity scaling, histogram equalization, or white-balance correction was applied. The preprocessed full image was passed in its entirety, without cropping, to both segmentation models: Mask R-CNN produced per-tooth crown instance masks, and YOLO11l-seg produced the gingival mask (inference confidence threshold 0.1). The resulting raw segmentation outputs then underwent model-specific post-processing prior to measurement extraction. Background masking was performed implicitly through the segmentation step itself, and no additional artifact-removal step was applied.

#### Mask post-processing

Raw segmentation outputs underwent model-specific post-processing prior to measurement extraction. Tooth crown masks from Mask R-CNN were converted to 8-bit binary masks using the framework-default mask threshold of 0.5(equivalently, a value of 127 on the 0–255 scale), resized to the input image dimensions by bilinear interpolation where required, and filtered by spatial overlap with the clinician-drawn bounding rectangle, with the central tooth identified as the mask located fully, or with ≥ 95% of its area, inside the rectangle. The central tooth and its left and right neighbours were then selected, sorted left-to-right by bounding-box x-coordinate, and each reduced to its largest connected component via contour-area selection. Gingival masks from YOLO11l-seg (inference performed at a detection confidence threshold of 0.1 to maximise candidate recall) were binarised, resized to the original image dimensions using nearest-neighbour interpolation to preserve binary edge sharpness, and combined into a single union mask by a pixel-wise OR, so that multiple disconnected gingival regions were treated as one anatomical structure for downstream analysis. No morphological filtering, hole filling, or contour smoothing was applied to the segmentation masks themselves, and no geometric transformations altering anatomical relationships were applied during test-time evaluation.

#### Morphological operations at measurement steps

Morphological operations were applied only at specific downstream measurement steps, never to the segmentation masks themselves, and two distinct operations were used. First, when two adjacent tooth masks did not intersect directly, iterative dilation (3 × 3 kernel, two iterations per pass, up to two successive passes) was applied to recover a contact region for papilla-apex extraction (Sect. Landmark definitions and extraction). Second, during the papilla recession check, a morphological opening with an elliptical 3 × 3 kernel was applied to the thresholded dark-region map to remove isolated noise before contour detection (Sect. Feature computation). No other morphological processing was performed.

#### Automated exclusion and failure-mode handling

During inference, all visibility and segmentation requirements are checked automatically and deterministically; no manual case review or selective exclusion is performed. When a per-attribute requirement is not met, the pipeline does not halt but assigns a deterministic conservative score of 0 for that attribute, consistent with a poor esthetic outcome (e.g., a papilla with no recoverable apex, or a gingival margin with no identifiable contralateral reference tooth). The remaining valid attribute sub-scores are still computed for that image. Because every step is rule-based and deterministic, identical inputs yield identical scores, and no manual selection enters the pipeline at any stage.

PES attribute scoring proceeded in three sequential phases: landmark extraction, feature computation, and threshold-based score assignment.

#### Landmark definitions and extraction

The rule-based pipeline operates on segmentation-derived geometric proxies rather than directly identified clinical anatomical landmarks. Each landmark referenced in the scoring rules is operationalised as a deterministic geometric construct computed from the post-processed tooth and gingival masks (Sect. Mask post-processing); no manual landmark placement or interactive refinement is performed. To restrict analysis to the maxillary arch, only teeth positioned above, or near, the vertical midpoint of the identified central tooth were retained for measurement. The principal landmarks are defined as follows:

- Papilla apex. The topmost point (minimum y in image coordinates) of the intersection zone between two adjacent tooth masks, computed by a pixel-wise AND of the masks followed by contour extraction and minimum-y selection. When the masks do not intersect at first instance, the apex is approximated via the iterative dilation fallback described in Sect. Morphological operations at measurement steps; if this fallback fails to produce an intersection after the maximum number of passes, apex extraction for that tooth pair is recorded as a failure and the corresponding papilla attribute is assigned a score of 0 without further geometric computation. The papilla apex also serves as the geometric proxy for the anatomical interdental contact point.
- Papilla baseline. The line segment connecting the rightmost top-edge point of the left tooth and the leftmost top-edge point of the right tooth, each taken at the corresponding crown’s minimum-y.
- Crown boundary extrema. The leftmost and rightmost x-coordinates at the minimum y across all contour points of each tooth crown. These anchor both the papilla triangle base and the gingival margin reference for that tooth.
- Gingival margin reference. The top-edge point of the tooth crown contour at the crown–gingiva interface, with its y-coordinate representing the gingival-margin height for that tooth. The anatomical gingival zenith is operationalised through this construct; no separate computation of the most apical curve of the marginal gingiva is performed.
- Mid-facial gingival level. Not extracted as a discrete landmark in this system; the gingival margin is approximated by the gingival pixel zone above the tooth mask, bounded above by the clinician-drawn bounding rectangle and below by the tooth’s crown apex.

For each adjacent tooth pair (left–central and central–right), these constructs were applied to derive the papilla apex and triangle baseline. For the gingival margin, the contralateral reference tooth was identified using the FDI symmetric-offset table (e.g., 11 ↔ 21, 12 ↔ 22), and its gingival-margin reference y-coordinate was extracted in the same way as for the central tooth.

#### Feature computation

For each papilla (mesial and distal), a binary recession flag was computed as the presence or absence of a dark contour within an apically displaced detection triangle inside the papilla region, with the thresholded dark-region map cleaned by the morphological opening described in Sect. Morphological operations at measurement steps before contour detection. A vertical fill ratio (r) was then computed as the relative height of the detected gingival contour within the papilla triangle, measured from base to apex:

r = (y_base − y_gingiva)/(y_base − y_apex).

where y_base is the y-coordinate of the papilla triangle base, y_apex is the y-coordinate of the papilla apex, and y_gingiva is the lowest visible gingival point (maximum y-coordinate) of the gingival union mask within the papilla triangle. By this construction, r increases as the gingival tissue extends further from the triangle base toward the apex, ranging from 0 (gingiva at the base, no fill) to 1 (gingiva reaching the apex, full fill). The fill ratio reflects the vertical position of the gingival contour within the papilla triangle and is not calculated from the proportion of triangle area occupied by gingival pixels.

For the gingival margin, the vertical pixel distance between the implant and contralateral gingival-margin y-coordinates was computed and normalised by the central tooth’s crown height, expressed as a percentage:

Percent distance = (|y_implant − y_contralateral|/h_crown_central) × 100%.

where y_implant and y_contralateral are the gingival-margin y-coordinates of the central (implant) and contralateral teeth, and h_crown_central is the vertical extent of the central tooth crown.

For gingival color, the region of interest above the implant crown was converted from BGR to HSV. If any single pixel in this region had an HSV Value below 70, the gingival-color attribute was assigned a score of 0; no CIELAB conversion or ΔE computation was performed. When this dark-pixel rule did not fire, the gingival-color score was assigned by a vision–language model (Qwen 2.5-VL).

#### Score assignment

Final attribute scores (0, 1, or 2) were assigned by threshold rules calibrated through the two-stage procedure described in Sect. Threshold calibration.

Papilla scoring was recession-gated, using uniform thresholds for the mesial and distal papilla: if no recession was detected, the score was 2; if recession was detected, the score was 1 when *r* ≥ 0.58 and 0 otherwise.

Gingival-margin thresholds were defined per tooth type. For central incisors (FDI 11/21): distance ≤ 5% of crown height → score 2; ≤ 10% → score 1; > 10% → score 0. For lateral incisors (FDI 12/22): ≤ 15% → score 2; ≤ 20% → score 1; > 20% → score 0. When the contralateral natural tooth cannot be identified from the segmentation output (e.g., an absent mask or out-of-frame anatomy), the gingival-margin attribute is assigned a conservative score of 0, and the remaining attribute sub-scores are still computed. Recording the attribute as missing data rather than defaulting to 0 is a planned refinement; in the present test set, a contralateral reference tooth was available for all images, so this pathway was not triggered.

Gingival color was scored by the dark-pixel rule followed by the vision–language fallback: any pixel with HSV Value < 70 in the implant-crown region set the score to 0; otherwise, Qwen 2.5-VL assigned a score of 0, 1, or 2.

For each photograph, the system generated four PES attribute scores (mesial papilla, distal papilla, gingival margin, and gingival color) and a total PES score in the range 0–8. Region processing and color-detection steps are illustrated in Fig. 6.

### Statistical analysis

Automated PES outputs were compared with expert reference scores. Diagnostic accuracy for individual PES attributes was evaluated using overall accuracy, 3 × 3 confusion matrices (ground truth versus predicted score class), and per-class metrics sensitivity (per-class recall), specificity, precision, and F1-score for each score class (0, 1, 2). Macro-averaged sensitivity, specificity, precision, and F1 were also computed per attribute to summarize performance across imbalanced classes. 95% confidence intervals for primary metrics were derived by non-parametric bootstrap resampling (2,000 resamples). Total PES performance was assessed using exact agreement and clinically relevant tolerance criteria ± 1 PES point.

Regression-based metrics, including mean absolute error (MAE), root mean squared error (RMSE), and coefficient of determination (R²), were calculated to assess agreement between automated and expert total PES scores. Statistical analyses were performed using Python-based scientific computing libraries.

## Results

### Test dataset and reference standard reliability

Of 865 postoperative intraoral photographs from 470 patients screened from the institutional archive (January 2020–June 2025), 475 were excluded during curation: most commonly implants outside the maxillary esthetic zone (*n* = 200) and duplicate images from the same patient, for which a single representative image was retained (*n* = 95); full reasons are given in Table 2. This yielded 390 eligible photographs from 390 unique patients. The 390 images were randomly allocated at the patient level into a development set of 308 images, used for segmentation-model training, internal validation, and fixing the rule-based scoring thresholds, and an independent test set of 82 images reserved for end-to-end evaluation. The tooth-crown segmentation model was trained separately on the external Teether dataset (1,966 images) and was never exposed to any cohort image. The 82 test photographs were therefore unseen by both segmentation models and were excluded from threshold setting, providing a leakage-free assessment. On the test set, the reference standard was the consensus of two calibrated periodontists who independently scored each photograph, with disagreements resolved through discussion. Inter-examiner reliability for the ordinal PES-derived attributes was assessed using weighted Cohen’s kappa, yielding a κ of 0.80, indicating substantial agreement.

The 390 included patients had a mean age of 44.3 ± 7.5 years and were predominantly female (270 [69.2%] female; 120 [30.8%] male; Table 1). Implant sites spanned the maxillary esthetic zone, most frequently the central incisors (FDI 11, *n* = 77; FDI 21, *n* = 73). The median time since prosthetic delivery was 21 months (IQR 13.5–28.5). Peri-implant soft-tissue status ranged from healthy to severe inflammation (healthy 40 [10.3%], mild 179 [45.9%], moderate 146 [37.4%], severe 25 [6.4%]), and peri-implant diagnoses were healthy in 82 (21.0%), mucositis in 163 (41.8%), and peri-implantitis in 145 (37.2%). Image quality was deliberately heterogeneous: 36 (9.2%) photographs were rated good, 163 (41.8%) acceptable, and 191 (49.0%) showed mild imperfections, with 191 containing challenging features (e.g., saliva, shadows, highlights, blur) and 88 containing multiple artefacts; reflecting real-world acquisition conditions.

### Attribute-level diagnostic performance

**Fig. 5:**
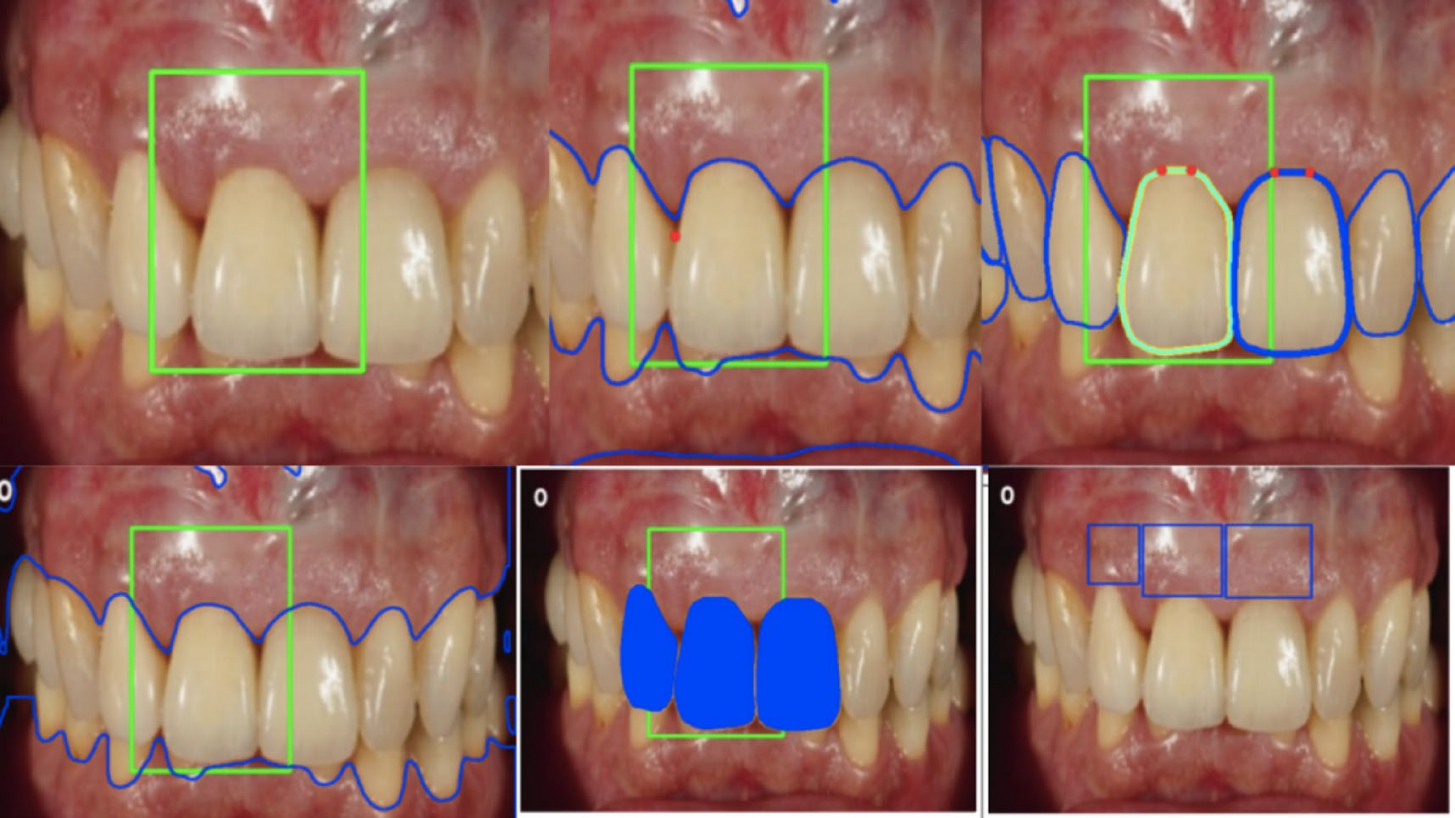
Per-attribute confusion matrices on the independent test set. Row-normalised 3 × 3 confusion matrices compare automated scores (columns) against the expert reference standard (rows) for each PES attribute: mesial papilla, distal papilla, gingival margin, and gingival color across the 82-image independent test set. Each cell gives the absolute count and the within-row (ground-truth) proportion. Diagonal dominance indicates agreement; for the distal papilla, misclassifications fell predominantly between the extreme categories (0 and 2) rather than between adjacent categories.

Across the four PES attributes, observed agreement between the automated system and the expert consensus ranged from 82.9% to 91.5% on the 82-image test set (Table 3a; per-class performance and confusion matrices in Table 4; Figure 5). All four attributes were imbalanced toward the healthy category, so accuracy is reported alongside macro-averaged metrics to avoid masking minority-class performance.

**Table 3:** Agreement between the automated Pink Esthetic Score (PES) system and the expert-consensus reference standard on the independent test set (n = 82).

(a) Per-component agreement					
PES component	Observed agreement	Unweighted κ	Linear weighted κ	Quadratic weighted κ (95% CI)	Reference distribution (0 / 1 / 2)
Mesial papilla	91.5%	0.86	0.84	0.83 (0.68–0.95)	20 / 19 / 43
Distal papilla	85.4%	0.70	0.59	0.48 (0.23–0.72)	9 / 17 / 56
Gingival margin	86.6%	0.75	0.74	0.74 (0.55–0.89)	10 / 20 / 52
Soft-tissue color	82.9%	0.49	0.55	0.61 (0.31–0.82)	6 / 11 / 65

(b) Total PES score agreement	
Total PES score (0–8) — metric	Value (95% CI)
Exact-match accuracy	57.3%
Accuracy within ±1 point	79.3%
Mean absolute error	0.76 points
Root mean squared error	1.35 points
Coefficient of determination (R^2^)	0.49
Cohen’s κ: unweighted	0.47
Cohen’s κ :linear weighted	0.59
Cohen’s κ: quadratic weighted	0.69 (0.50–0.82)
ICC two-way, absolute agreement, single measures	0.69 (0.56–0.79)
Bland–Altman bias (mean difference)	+0.07 (SD 1.35)
95% limits of agreement (LoA)	−2.59 to +2.74 points
Proportional-bias slope (difference vs. mean)	−0.28 (p = 0.003)
Regression slope (automated vs. reference)	0.56

Bland–Altman difference = automated − reference; limits of agreement = bias ± 1.96 SD. PES = Pink Esthetic Score; κ = Cohen’s kappa; CI = confidence interval; ICC = intraclass correlation coefficient; SD = standard deviation.

**Table 4:** Confusion matrices and class-wise diagnostic performance for automated PES component scoring (independent test set, n = 82).

(a) Mesial papilla : overall accuracy 91.5%				
Reference \ Predicted	Score 0	Score 1	Score 2	Row total
Score 0	17 (85.0%)	0	3 (15.0%)	20
Score 1	0	16 (84.2%)	3 (15.8%)	19
Score 2	1 (2.3%)	0	42 (97.7%)	43
Class	Sensitivity / Recall	Specificity	Precision	F1-score
Score 0	0.850	0.984	0.944	0.895
Score 1	0.842	1.000	1.000	0.914
Score 2	0.977	0.846	0.875	0.923
Macro avg	0.890	0.943	0.940	0.911

(b) Distal papilla : overall accuracy 85.4%				
Reference \ Predicted	Score 0	Score 1	Score 2	Row total
Score 0	5 (55.6%)	0	4 (44.4%)	9
Score 1	0	15 (88.2%)	2 (11.8%)	17
Score 2	6 (10.7%)	0	50 (89.3%)	56
Class	Sensitivity / Recall	Specificity	Precision	F1-score
Score 0	0.556	0.918	0.455	0.500
Score 1	0.882	1.000	1.000	0.938
Score 2	0.893	0.769	0.893	0.893
Macro avg	0.777	0.896	0.783	0.777

(c) Gingival margin : overall accuracy 86.6%				
Reference \ Predicted	Score 0	Score 1	Score 2	Row total
Score 0	5 (50.0%)	4 (40.0%)	1 (10.0%)	10
Score 1	1 (5.0%)	18 (90.0%)	1 (5.0%)	20
Score 2	2 (3.8%)	2 (3.8%)	48 (92.3%)	52
Class	Sensitivity / Recall	Specificity	Precision	F1-score
Score 0	0.500	0.958	0.625	0.556
Score 1	0.900	0.903	0.750	0.818
Score 2	0.923	0.933	0.960	0.941
Macro avg	0.774	0.932	0.778	0.772

(d) Soft-tissue color :overall accuracy 82.9%				
Reference \ Predicted	Score 0	Score 1	Score 2	Row total
Score 0	4 (66.7%)	0	2 (33.3%)	6
Score 1	2 (18.2%)	4 (36.4%)	5 (45.5%)	11
Score 2	1 (1.5%)	4 (6.2%)	60 (92.3%)	65
Class	Sensitivity / Recall	Specificity	Precision	F1-score
Score 0	0.667	0.961	0.571	0.615
Score 1	0.364	0.944	0.500	0.421
Score 2	0.923	0.588	0.896	0.909
Macro avg	0.651	0.831	0.656	0.649

(e) Macro-averaged summary across attributes					
Attribute	Accuracy	Sensitivity	Specificity	Precision	Macro F1
Mesial papilla	0.915	0.890	0.943	0.940	0.911
Distal papilla	0.854	0.777	0.896	0.783	0.777
Gingival margin	0.866	0.774	0.932	0.778	0.772
Soft-tissue color	0.829	0.651	0.831	0.656	0.649

Agreement was highest for the mesial papilla (91.5%; quadratic weighted κ = 0.83, 95% CI 0.68–0.95), with near-perfect recovery of the most prevalent category (Score 2 recall 0.977) and balanced performance across the minority categories (Score 0 recall 0.850; Score 1 recall 0.842; macro-F1 0.911). The gingival margin showed similarly strong agreement (86.6%; quadratic weighted κ = 0.74, 0.55–0.89), driven by high recall for Score 2 (0.923), with the main errors in the under-represented Score-0 category (recall 0.500; 10 reference cases).

The distal papilla reached comparable raw agreement (85.4%) but a substantially lower quadratic weighted κ (0.48, 0.23–0.72). This dissociation arose because its misclassifications fell predominantly between the two extreme categories rather than between adjacent ones: of nine reference Score-0 cases, four (44%) were automatically scored 2 (Score-0 recall 0.556), and six of 56 reference Score-2 cases were scored 0 (Figure 5, confusion matrices). Because quadratic weighting penalises such two-step (0-vs-2) disagreements most heavily, the weighted κ was attenuated despite high overall agreement, and the macro-F1 (0.777) fell well below the accuracy.

**Fig. 6:**
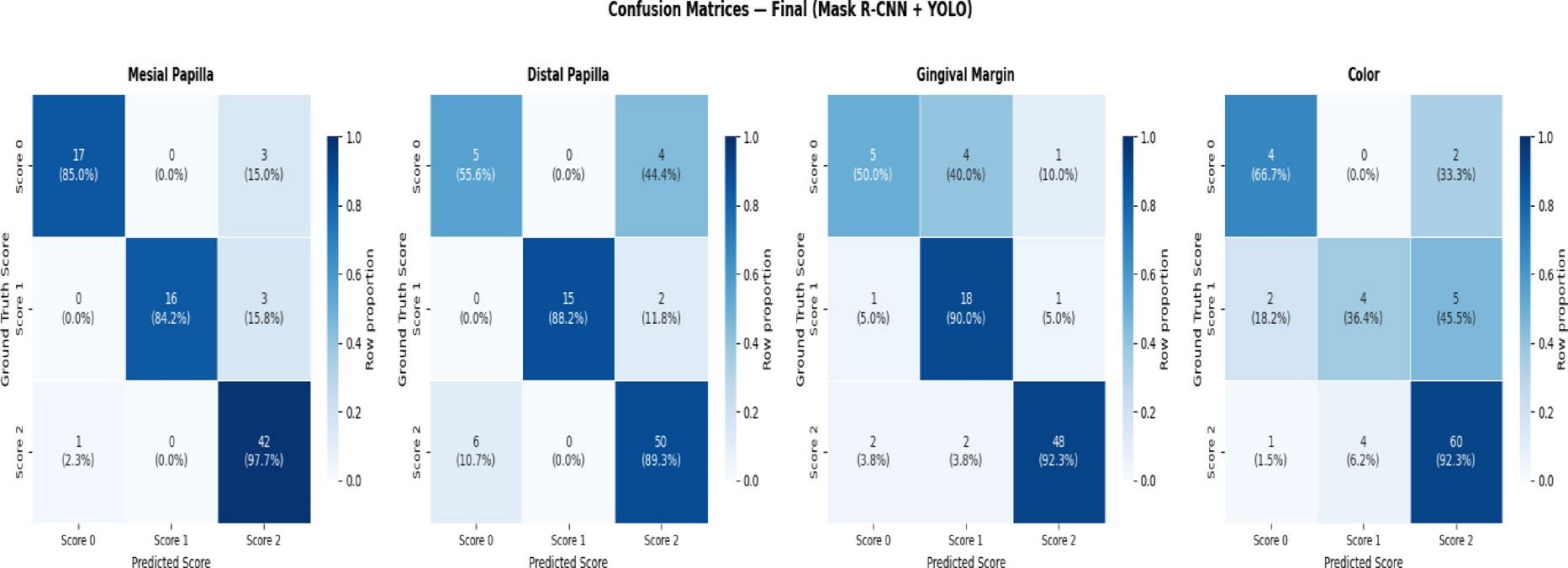
Representative qualitative output of the segmentation and landmark-extraction stages on a test-set photograph. The working tooth is first localised within its bounding box (green) from the supplied FDI label (**a**), constraining all downstream measurements to the correct implant site. The gingival margin is then delineated by YOLO11l-seg (**b**), and the tooth-crown contour segmented by Mask R-CNN is overlaid with the gingival outline to locate the anatomical landmarks used for scoring, papilla apices, and gingival reference points (red) (**c**). Lower panels show the gingival contour (**d**), the tooth-crown mask (**e**), and the papilla regions of interest (**f**) from which the mesial and distal papilla attributes are derived.

Agreement for soft-tissue color was 82.9% (quadratic weighted κ = 0.61, 0.31–0.82). Here, the weighted κ exceeded the unweighted value (0.49), indicating that disagreements were predominantly adjacent: the minority Score-1 category was the most difficult (recall 0.364; misassigned to the neighbouring Score 0 or Score 2), whereas the dominant Score-2 category was recovered reliably (recall 0.923). The skewed reference distribution for this attribute attenuates the unweighted κ and produces the largest macro-vs-accuracy gap of the four attributes (macro-F1 0.649 vs. accuracy 0.829) (Fig. 6).

### Agreement analysis for total pink esthetic score

**Fig. 7:**
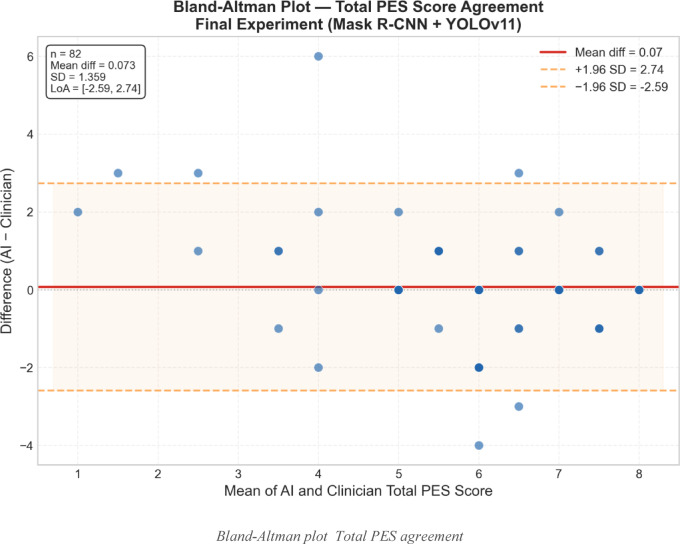
Bland–Altman analysis of total Pink Esthetic Score (PES) agreement between the automated system and the expert-consensus reference standard (independent test set, *n* = 82).

**Fig. 8:**
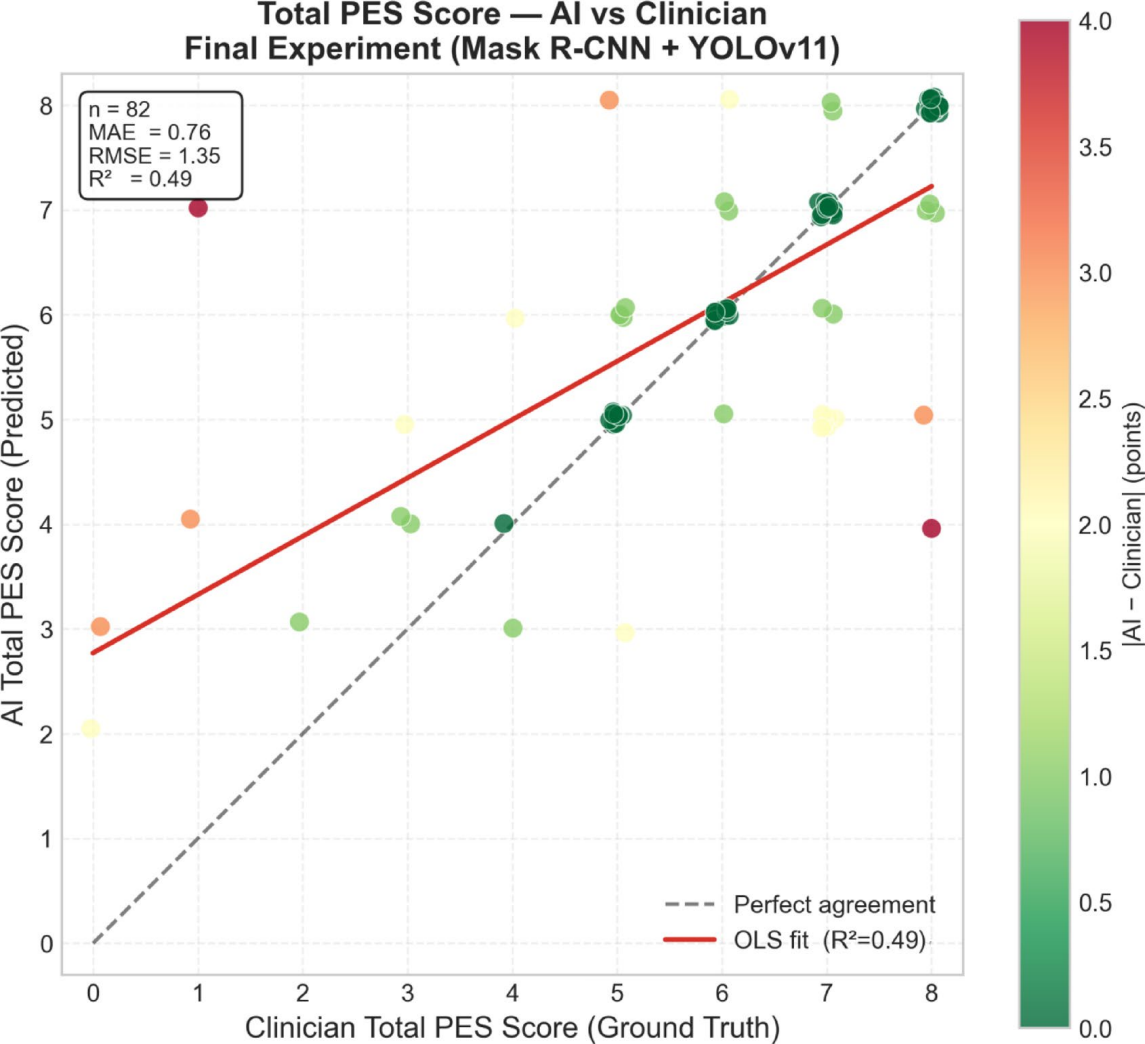
Automated versus reference total PES (independent test set, *n* = 82). Each point is one case, plotted as the automated total PES (y-axis) against the expert-consensus reference total PES (x-axis) and color-coded by absolute error. The dashed line is the line of perfect agreement (identity); the solid line is the ordinary-least-squares fit (slope = 0.56, R² = 0.49). Total PES scatter AI vs Clinician.

For the summed PES (range 0–8), the automated total matched the expert reference exactly in 57.3% of cases and to within ± 1 point in 79.3% (Table 3b, agreement), with a mean absolute error of 0.756 points and a root-mean-squared error of 1.352 points. Because the total is an ordinal sum, agreement was quantified primarily by Cohen’s quadratic weighted κ = 0.69 (0.50–0.82) and the intraclass correlation coefficient (ICC = 0.69, 0.56–0.79; two-way random-effects, absolute-agreement, single measures); the unweighted κ was 0.47, the gap reflecting that the great majority of discrepancies were single-step rather than large. Bland–Altman analysis showed negligible mean bias (+ 0.07 points; 95% limits of agreement − 2.59 to + 2.74; Figure 7, Bland–Altman). As a secondary, descriptive check for systematic bias, regression of the case-level difference on the case mean revealed a small but significant proportional bias (slope − 0.28, *p* = 0.003), and regression of automated on reference scores (slope = 0.56, R² = 0.49; Figure 8, predicted vs. reference) confirmed mild compression toward mid-range values, with the system tending to over-score low-PES and under-score high-PES cases (Table 4).

## Discussion 

The present study evaluated an anatomy-driven hybrid deep learning framework for automated photographic assessment of PES attributes. **T**his work is positioned as a method development and validation study, intended to establish the feasibility and reference-standard agreement of the proposed pipeline rather than to characterise performance across deployment conditions or clinical subpopulations. The principal finding is that when explicit anatomical segmentation precedes scoring, automated evaluation of PES attributes can achieve clinically meaningful agreement with expert assessment while maintaining interpretability and structural transparency.

To the authors’ knowledge, this is the first deep learning model to attempt predefined rule-based criteria to derive esthetic scores from measurable attributes. The system was intentionally designed to operate on a single frontal intraoral photograph to reflect the conventional clinical assessment of the PES, which is typically performed from a standardized frontal view.

The final architecture was selected empirically rather than assumed. Four pipeline configurations were constructed by pairing the two instance-segmentation frameworks (Mask R-CNN and YOLO11l-seg) across the tooth-crown and gingival segmentation tasks, and each was evaluated by its agreement with expert PES scores on a labelled portion of the training set. The combination of Mask R-CNN (ResNet-50–FPN) for tooth-crown segmentation and YOLO11l-seg for gingival segmentation produced the closest agreement and was therefore adopted. The framework is, by design, hybrid: deep-learning computer vision supplies the anatomical structures, while a deterministic rule-based layer converts these into ordinal PES attributes, with a vision–language model invoked only for ambiguous color cases. This pairing combines the perceptual strength of learned segmentation with the transparency of explicit logic, so that each attribute score can be traced to a defined geometric criterion rather than an opaque end-to-end prediction.

The two segmentation models were trained on deliberately different datasets, each matched to the demands of its task. Tooth-crown segmentation operates on high-contrast, geometrically stable structures that transfer reliably across acquisition conditions and was therefore trained on a large, heterogeneous external dataset, whereas gingival segmentation is far more sensitive to tissue color, contour, and lighting and was trained on domain-matched in-house photographs acquired under the study’s own protocol. Because errors in an upstream stage propagate to downstream stages in cascaded imaging pipelines^17^, and because the geometric measurements depend directly on mask quality, strengthening each segmentation step with task-appropriate data limits the upstream segmentation error available to propagate into landmark extraction and scoring. This propagation is further limited by the scoring design, in which the gingival margin is referenced to the contralateral tooth and the papilla to adjacent-crown contact points, so that systematic boundary deviations affecting both structures tend to cancel rather than accumulate.

Both segmentation models were trained using standard, well-established configurations rather than parameters tuned to maximise agreement on the present dataset; this level of performance was therefore obtained without extensive hyperparameter optimisation. This choice favours reproducibility and portability and suggests that the observed accuracy reflects the robustness of the segmentation-and-scoring framework itself rather than dataset-specific tuning, consistent with evidence that, for many machine-learning algorithms, default configurations perform competitively and large gains from exhaustive tuning are not generally to be expected^18^. It also implies headroom for further improvement: targeted optimisation of training schedules, augmentation, or backbone capacity could raise segmentation quality, and hence downstream scoring, without altering the underlying rule-based design.

While the segmentation models were trained with standard configurations, the rule-based scoring layer depends on a small set of decision thresholds that translate each continuous geometric feature into an ordinal PES category. To avoid arbitrary cutoffs, a threshold sweep over candidate values was performed on a labelled portion of the training data, selecting the thresholds that best separated adjacent categories across all score classes so that an imbalanced distribution would not be masked by the majority class; adjusting decision thresholds in this way is an established strategy for handling class imbalance without retraining or resampling^19^. The resulting cutoffs were then frozen and applied unchanged to every test image. Calibrating the thresholds on labelled training data in this way, separately from, and prior to, evaluation, addresses class imbalance at the decision boundary itself while keeping the scoring rule explicit and fully deterministic.

In this system, the inference stage is fully deterministic: the pipeline runs as a single pass in which each photograph is segmented, the geometric features are measured, and the pre-calibrated thresholds are applied to assign the four attribute scores, with no parameters learned or adjusted at test time. Crucially, the scoring stage carries very few free parameters: a single fill-ratio cutoff and the per-tooth-type gingival-margin percentages, so the mapping from measurements to PES categories has limited capacity to memorise idiosyncrasies of the data, in contrast to a high-capacity end-to-end regressor; this accords with the argument that interpretable, low-complexity models are preferable to opaque high-capacity models for high-stakes decisions^20^. Because every decision rule is fixed and explicit, identical inputs yield identical outputs, and no manual case review or selective exclusion enters the process; when a required landmark cannot be recovered, a deterministic conservative score is assigned rather than the case being discarded.

At the attribute level, the mesial papilla demonstrated the highest accuracy, followed by the gingival margin and the distal papilla, consistent with previous studies reporting on the anatomical clarity of papillary landmarks in standardized frontal intraoral photographs^21^. Gingival color assessment yielded the lowest accuracy, in line with previous studies highlighting the sensitivity of color-based analysis to light-source variability, camera settings, and soft-tissue texture^22^^[,23^, underscoring the importance of cautious interpretation of color-related outcomes in photographic AI applications. The distal papilla warrants separate comment: despite high overall accuracy (85.4%), it showed the lowest quadratic weighted κ of the four attributes (0.48), because its misclassifications fell predominantly between the extreme categories (Score 0 versus Score 2) rather than between adjacent ones.

Confusion-matrix analysis showed that, for the mesial papilla, gingival margin, and gingival color, misclassifications occurred mainly between adjacent PES scores, so these discrepancies reflected subtle esthetic gradations rather than clinically misleading errors. The distal papilla was the exception, with errors concentrated between the extreme categories; because quadratic weighting penalises such two-step disagreements most heavily, its weighted κ was attenuated despite high raw agreement. Exact agreement for total PES was observed in 57.3% of cases, increasing to 79.3% within a clinically acceptable tolerance of ± 1 point; given the ordinal structure and documented inter-examiner variability of esthetic indices¹¹, this level of agreement is consistent with expected scoring behaviour. Regression metrics indicate moderate correspondence across the scoring range, while the aggregation of multiple ordinal attributes into a composite score likely amplifies minor attribute-level deviations, explaining the difference between high attribute-level accuracy and moderate total-score exact agreement.

The results of this study differ from previous CNN-based studies^24,25^, which reported limited performance for multi-class assessments and only moderate performance for binary classifications. This divergence likely reflects methodological differences, as prior approaches relied on end-to-end classification without explicit anatomical modeling. In contrast, the present study implemented a hybrid pipeline combining dedicated segmentation backbones for tooth crown and gingival delineation with rule-based, geometry-driven scoring. Such a structured, measurement-oriented framework may offer improved stability and closer alignment with clinical reasoning compared with purely predictive classifier models.

This interpretation is supported by comparison with a previous study that employed a deep learning regression approach using multiple photographic views and clinical variables to predict total postoperative PES^15^. While that model demonstrated acceptable accuracy, 65.93%, it operated as an implicit prediction system. In contrast, the present approach decomposes the task into anatomically grounded subattributes, mirroring conventional clinical evaluation from a single standardized frontal photograph. This structured design may improve reproducibility in research contexts where standardized documentation is critical.

Error analysis indicated that most discrepancies were driven by anatomical variability rather than model instability. In cases of diastema or reduced proximal contact, the absence of a clear intersection point destabilized papilla triangle construction, introducing uncertainty in apex detection and fill ratio estimation. Minor segmentation boundary deviations in crowded regions propagated into papilla geometry calculations, particularly near threshold boundaries. Gingival margin differences occurred in asymmetrical crown morphologies or when contralateral reference teeth were non-ideal due to crown-height normalization. Pink porcelain restorations and non-uniform illumination also influenced color assessment. Importantly, most deviations remained within ± 1 of expert grading, reflecting sensitivity near decision thresholds rather than structural failure of anatomical interpretation. The deliberate asymmetry between the externally sourced tooth segmentation dataset and the in-house gingival dataset reflects both the differing domain sensitivity of the two segmentation tasks and the practical absence of any publicly available peri-implant gingival segmentation resource at the time of this work. Residual tooth-segmentation errors, including those potentially attributable to domain mismatch, propagate most strongly in anatomically complex cases (diastema, crowding, asymmetric crown morphology), where they intersect with the geometric assumptions of papilla triangle construction.

Several limitations should be acknowledged. The dataset was restricted to postoperative frontal photographs of single-tooth implants in the maxillary esthetic zone from a single-center population, which may limit external generalizability. The present work is a method development and validation study; its primary objective was to establish agreement of the automated PES system against a clinical reference standard rather than to characterise performance across clinical subpopulations. Accordingly, the evaluation set was a random, leak-proof hold-out that was not stratified by image quality, implant site, gingival phenotype, or restoration type, and the resulting subgroups are too small and unevenly populated to support reliable stratified estimates. Adequately powered, prospectively stratified subgroup analysis with sampling stratified by PES distribution and implant site is a priority for future multi-centre validation work, where pre-stratification becomes both feasible and methodologically advantageous given larger sample sizes and site-level heterogeneity. Exclusion of technically inadequate photographs at the curation stage may additionally have introduced a curation bias toward well-visualised cases; performance under the lower-quality end of routine clinical photography, therefore, warrants prospective evaluation. As a single-centre retrospective dataset, complete blinding to case familiarity cannot be guaranteed for senior clinicians who may have been involved in the original clinical care, although no clinical case information was actively presented during scoring.

The present framework assessed four PES-derived attributes, as the contour attribute was excluded due to the inherent limitations of standardized two-dimensional photographic records in reliably capturing three-dimensional soft tissue convexity. Consequently, the system does not currently represent the full original PES index.

Segmentation masks were generated by a single trained annotator following standardised written annotation guidelines under expert supervision. Although calibration procedures and consensus-developed annotation protocols were implemented, the resulting labels may still reflect a single annotator’s boundary interpretation style rather than a robust multi-annotator consensus, and the model may partially learn annotator-specific segmentation behaviour, particularly in anatomically ambiguous regions.

Relatedly, the polygon annotator also served as one of the two reference-standard PES scorers; however, three methodological features mitigate this overlap. First, PES scoring was performed independently by a senior periodontist co-author in addition to the first author, with disagreements resolved by consensus, so the first author’s individual scoring style cannot unilaterally define the reference. Second, both scorers were blinded to AI outputs throughout. Third, polygon mask annotation (drawing pixel-level tissue boundaries) and PES attribute scoring (assigning ordinal categories from raw photographs) are cognitively distinct tasks operating on different inputs and outputs, so circulation bias is at most partial rather than direct. Nonetheless, future studies should incorporate multiple independent annotators with different subspecialty backgrounds and compute formal inter-annotator agreement analysis and consensus-based mask refinement.

In this study, formal segmentation performance was also not quantified at the mask level: Dice coefficient, IoU, precision, recall, and boundary-aware measures were not computed against ground-truth masks for either segmentation model, and segmentation quality was assessed only indirectly through downstream agreement of the end-to-end PES scores with the expert reference and through comparison of segmentation-backbone combinations by their effect on that scoring. Direct mask-level evaluation against retained ground-truth annotations, including boundary-aware metrics for any future extension that relies on dense contour geometry, is a requirement for future work.

The rule-based system operates on segmentation-derived geometric proxies rather than directly identified anatomical landmarks; deviations between these proxies and their true anatomical referents, such as the gingival zenith and mid-facial gingival level, may contribute to attribute-level scoring error, particularly in cases with non-standard crown morphology or asymmetric papilla architecture. The current system also produces only discrete categorical scores; cases in which the underlying continuous measurement falls near a scoring threshold are not distinguished from clear-cut cases. Implementing a margin-based flagging mechanism that escalates such borderline cases for clinician verification, for example, by surfacing cases within a small tolerance of the papilla, gingival margin, or color thresholds, represents an important direction for future clinical deployment.

Postoperative photographs were acquired during routine clinical care without a color-calibration target (e.g., ColorChecker chart) in the scene. The system mitigates this through per-image self-normalisation, but retains residual sensitivity to severe color casts and extreme illumination artefacts, which may bias the gingival color attribute in such cases. Prospective validation studies should consider including a color-calibration target in the photographic protocol to enable explicit color correction and to assess system performance on calibrated imagery.

Finally, a specific random seed was not enforced during training of the Mask R-CNN tooth segmentation model, and inter-run variability across the two segmentation networks was not formally quantified; multi-seed variability quantification is recommended for future prospective and multi-centre extensions. Robustness under systematic image perturbations such as brightness and contrast shifts, blur, and cropping was not evaluated, and quantifying performance under such photometric and geometric degradations remains an objective for future work. Relatedly, the system was evaluated only on images in which the peri-implant anatomy was visible, and the case was scorable; a deployable implementation will require an automated mechanism to detect and flag genuinely unscorable images, such as those lacking the necessary anatomical visibility, so that they are referred for re-capture rather than scored unreliably.

Future research should include multicentre external validation, longitudinal performance assessment, integration with three-dimensional imaging modalities to enable evaluation of soft tissue contour, refinement of color normalisation techniques, and direct mask-level segmentation evaluation against retained ground-truth annotations. Exploration of multimodal inputs and standardised photographic calibration protocols may further enhance robustness and clinical applicability across diverse patient populations and imaging conditions.

## Conclusion

The findings of this study suggest that an anatomy-driven hybrid segmentation framework may provide a structured approach for automated photographic assessment of PES attributes. By integrating anatomical segmentation with rule-based measurement and statistically derived threshold sweeping, the proposed system offers a transparent alternative to end-to-end predictive models for esthetic evaluation.

While further external validation is required, this framework may serve as a foundation for the development of reproducible and interpretable AI-supported tools for standardized peri-implant esthetic assessment in research settings.

## Supplementary Information

Below is the link to the electronic supplementary material.

Supplementary Material 1

## Data Availability

The data set is not publicly available due to patient privacy considerations, but is available from the corresponding author upon reasonable request.
