# Supplementary material for: Artificial intelligence-based photographic detection of pink esthetic score attributes using a hybrid deep learning segmentation pipeline: a method development study
Source: Sci Rep. 2026 Aug 1;16:23726. doi: 10.1038/s41598-026-62229-4 (PMC13428750; doi:10.1038/s41598-026-62229-4)
Supplement: Supplementary file 1 — Supplementary Material 1 [file 41598_2026_62229_MOESM1_ESM.docx]

**1. Purpose**

Prior to dataset annotation, a standardized annotation protocol was developed to ensure consistent delineation of peri-implant soft tissue structures for training the gingival segmentation model. All annotations would be performed using polygon-based labeling in the Computer Vision Annotation Tool (CVAT).

**2. Annotated Classes**

**Four anatomical classes would be annotated:**

1. **Marginal Gingiva**
2. **Mesial Papilla**
3. **Distal Papilla**
4. **Alveolar Mucosa**

Each class would be annotated as an independent polygon object.

**3. General Annotation Rules**

- Annotation would be performed on the full uncropped image.
- Polygon annotation would be used exclusively.
- Freehand contours, brush masks, and bounding-box-assisted annotations would not be used.
- Polygon vertices would be placed directly on visible anatomical boundaries.
- Sufficient vertex density would be used to accurately represent curved tissue contours.
- Straight tissue borders would be annotated using fewer vertices to avoid unnecessary complexity.
- Polygon borders would not be artificially smoothed after annotation.

**4. Marginal Gingiva**

**Definition**

The marginal gingiva would be defined as the keratinized peri-implant soft tissue extending coronally around the implant-supported crown.

**Boundary Rules**

- The coronal border would follow the visible gingival margin adjacent to the implant crown.
- The apical border would correspond to the visible transition between keratinized gingiva and alveolar mucosa.
- Tooth structure would be excluded.
- Restorative material would be excluded.
- The entire visible marginal gingival contour would be included.

**5. Mesial Papilla**

**Definition**

The mesial papilla would be defined as the interdental soft tissue located between the implant-supported crown and the adjacent natural tooth on the mesial aspect (nearest to the midline).

**Boundary Rules**

- The papilla would be annotated as a separate structure from the marginal gingiva.
- The most coronal visible papillary peak would be included.
- Tooth structure and restorative material would be excluded.
- The base of the papilla would be defined at the point where the papillary contour merged with adjacent gingival tissues.

**6. Distal Papilla**

**Definition**

The distal papilla would be defined as the interdental soft tissue located between the implant-supported crown and the adjacent natural tooth on the distal aspect (nearest to the midline).

**Boundary Rules**

- Annotation would follow the same principles applied to the mesial papilla.
- The complete visible papillary contour would be included.
- Tooth structure and restorative material would be excluded.

**7. Alveolar Mucosa**

**Definition**

The alveolar mucosa would be defined as the non-keratinized soft tissue located apical to the marginal gingiva.

**Boundary Rules**

- Annotation would extend from the visible mucogingival junction to the visible limit of the image.
- Lip tissue and non-oral structures would be excluded.
- Areas obscured by retractors would not be inferred or reconstructed.

**8. Handling Ambiguous Regions**

**Specular Reflections**

- Visible tissue boundaries would be followed despite the presence of reflections.
- Reflection artefacts themselves would not be annotated as separate structures.

**Shadowed Regions**

- Mild shadowing would be retained when tissue boundaries remained identifiable.
- Severely obscured regions would not be inferred when anatomical boundaries could not be determined reliably.

**Partial Occlusion**

- Tissue obscured by retractors, instruments, saliva accumulation, or image artefacts would not be reconstructed.
- Only directly visible anatomy would be annotated.

**Tooth–Tissue Interfaces**

- Polygon borders would be placed along the visible interface between tooth structure and soft tissue.
- Tooth pixels would be excluded from all gingival classes.

**9. Quality Control and Calibration**

Before the commencement of full-dataset annotation, a calibration exercise would be conducted using 20 photographs that would not subsequently be included in either model development or testing.

The annotator's initial polygons would be reviewed with senior periodontology supervisors and members of the AI development team. Revisions and discussion would continue until consistent application of the protocol was achieved.

Following completion of calibration, all dataset annotations would be performed according to the finalized protocol and written guidelines.
